# Supplementary material for: Anaerobic bacterial degradation of protein and lipid macromolecules in subarctic marine sediment
Source: ISME J. 2020 Nov 18;15(3):833–47. doi: 10.1038/s41396-020-00817-6 (PMC8027456; doi:10.1038/s41396-020-00817-6)
Supplement: Supplementary file 1 — Supplementary information [file 41396_2020_817_MOESM1_ESM.docx]

**Supplementary information:**

**Anaerobic bacterial degradation of protein and lipid macromolecules in subarctic marine sediment**

**Supplementary materials and methods**

***Microbial community analysis of Svalbard samples***

Sediment samples from Smeerenburgfjorden, Svalbard, were collected in June 2017. The samples were taken with a HAPS corer from the vessel ‘MS Farm’ from station GK (79°38.49N 11°20.96E), and station J (79°42.83N 11°05.10E). The HAPS cores were subsampled on deck with a plastic subcorer that had 5 cm width and pre-drilled ports. Subsamples (1 mL of sediment) were taken with sterile 3 ml syringe, added to 2 ml tubes and flash frozen in a dry-shipper pre-cooled by liquid nitrogen. Sample were stored at -80°C in the laboratory until further analyses.

Extractions of RNA/DNA were performed with the RNeasy PowerSoil Total RNA Kit (Qiagen) according to manufacturer’s protocol. From the eluted nucleic acids, DNA for PCR was used as eluted. Aliquots of RNA extracts were DNase treated with the TURBO DNA-free kit (Thermo Fisher) following the manufacturer’s protocol. RNA was reverse transcribed using the Revert Aid First Strand cDNA Synthesis Kit (Thermo Fisher) following the manufacturer’s protocol. A reverse transcription negative control was performed by combining the remaining supernatant of the samples and adding all reagents except the Revert Aid M-MuLV Reverse Transcriptase. The cDNA was checked by PCR with primers targeting bacterial 16S rRNA genes and gel electrophoresis. The reverse transcription-negative control was always negative.

For 16S rRNA gene amplicon sequencing, a two-step PCR and barcoding approach was used [[1]](https://paperpile.com/c/rIw3WY/hY0at). The primers 515F (5′-GTGYCAGCMGCCGCGGTAA-3′) and 806R (5′-GGACTACNVGGGTWTCTAAT-3′) were used in the first step, and both primers included a linker sequence to facilitate barcoding in the second-step PCR. All first-step PCRs were performed in triplicates (and combined after PCR for barcoding) with a reaction volume of 12.5 µl. Each reaction (12.5µl) contained: 1X Dream Taq Buffer (including 2 mM MgCl_2_) (Thermo Fisher), 0.2 mM dNTP mix (Thermo Fisher), 0.2 µM of each forward and reverse primer, 0.08 mg ml^-1^ BSA (Thermo Fisher), 0.02 U Dream Taq Polymerase (Thermo Fisher), UV-treated deionised water (up to 12 µl) and 0.5 µl of template. PCR cycling was: 95°C for 3 min, followed by 30 cycles of 30 sec denaturation at 95°C, 30 sec annealing at 52°C and 50 sec elongation at 72°C, and a final 10 min at 72°C. Sequencing was performed by the Joint Microbiome Facility (Vienna, Austria) using an Illumina MiSeq with MiSeq Reagent Kit v3 chemistry with 300 bp paired-end read mode. Bioinformatic processing of 16S rRNA gene amplicon data from Svalbard sediments was performed by demultiplexing amplicon sequencing variants (ASVs) that were constructed using DADA2 [[2]](https://paperpile.com/c/rIw3WY/62j0Y) with the previously described workflow [[3]](https://paperpile.com/c/rIw3WY/ESatt).

**Supplementary discussion**

In addition to the relatively abundant taxa described above that were ^13^C-enriched and that could be linked to genomes or MAGs, we also found ^13^C-enrichment in several less-abundant taxa (<1% relative abundances in microcosms). Members of the *Fusibacter* (OTU 38) and *Photobacterium* (OTU 54) were determined to be ^13^C-labelled from protein incubations, while a *Vibrio* species (OTU 80) was determined to be ^13^C-labelled from lipid incubations. The *Photobacterium* (OTU 54) and *Vibrio* (OTU 80) also showed increased relative abundances in microcosms that were also specific to protein or lipid amended microcosms, respectively. This supported their roles in primary hydrolysis of these substrates, or had specific associations with primary hydrolyzers of either substrate. Members of these groups have been previously described to have relatively diverse heterotrophic metabolisms. For instance, close relatives of *Photobacterium* OTU 54 were previously shown to be both lipolytic [[4, 5]](https://paperpile.com/c/rIw3WY/zLOkr+6QoIt) and proteolytic [[6, 7]](https://paperpile.com/c/rIw3WY/AGqNd+AH1mn). The majority of OTU 80 related *Vibrio* *spp*. are able to degrade sugars and glycerol, and also include lipolytic isolates [[8–13]](https://paperpile.com/c/rIw3WY/te10W+b5Yeo+L2d0c+QEvXK+BLMTE+GG3l9). These data therefore suggest that these taxa may have played roles in primary hydrolysis of the macromolecules. In comparison, the *Fusibacter* (OTU 38) gradually increased in relative abundances in all microcosms including no-substrate controls, thereby suggesting they may have rather utilized fermentation products or degradation products common to the different treatments, e.g., acetate.

**Supplementary references**

1. [Herbold CW, Pelikan C, Kuzyk O, Hausmann B, Angel R, Berry D, et al. A flexible and economical barcoding approach for highly multiplexed amplicon sequencing of diverse target genes. *Frontiers in Microbiology* . 2015. , **6**](http://paperpile.com/b/rIw3WY/hY0at)

2. [Callahan BJ, McMurdie PJ, Rosen MJ, Han AW, Johnson AJA, Holmes SP. DADA2: High-resolution sample inference from Illumina amplicon data. *Nat Methods* 2016; **13**: 581–583.](http://paperpile.com/b/rIw3WY/62j0Y)

3. [Callahan BJ, Sankaran K, Fukuyama JA, McMurdie PJ, Holmes SP. Bioconductor Workflow for Microbiome Data Analysis: from raw reads to community analyses. *F1000Res* 2016; **5**: 1492.](http://paperpile.com/b/rIw3WY/ESatt)

4. [Seo HJ. Photobacterium aplysiae sp. nov., a lipolytic marine bacterium isolated from eggs of the sea hare Aplysia kurodai. *Int J Syst Evol Microbiol* 2005; **55**: 2293–2296.](http://paperpile.com/b/rIw3WY/zLOkr)

5. [Yoon J-H, -H. Yoon J. Photobacterium lipolyticum sp. nov., a bacterium with lipolytic activity isolated from the Yellow Sea in Korea. *Int J Syst Evol Microbiol* 2005; **55**: 335–339.](http://paperpile.com/b/rIw3WY/6QoIt)

6. [Zhang X-Y, Han X-X, Chen X-L, Dang H-Y, Xie B-B, Qin Q-L, et al. Diversity of cultivable protease-producing bacteria in sediments of Jiaozhou Bay, China. *Front Microbiol* 2015; **6**: 1021.](http://paperpile.com/b/rIw3WY/AGqNd)

7. [Li Y, Zhou M, Wang F, Wang ET, Du Z, Wu C, et al. Photobacterium proteolyticum sp. nov., a protease-producing bacterium isolated from ocean sediments of Laizhou Bay. *Int J Syst Evol Microbiol* 2017; **67**: 1835–1840.](http://paperpile.com/b/rIw3WY/AH1mn)

8. [Lucena T, Ruvira MA, Arahal DR, Macián MC, Pujalte MJ. Vibrio aestivus sp. nov. and Vibrio quintilis sp. nov., related to Marisflavi and Gazogenes clades, respectively. *Syst Appl Microbiol* 2012; **35**: 427–431.](http://paperpile.com/b/rIw3WY/te10W)

9. [Shieh WY. Vibrio ruber sp. nov., a red, facultatively anaerobic, marine bacterium isolated from sea water. *Int J Syst Evol Microbiol* 2003; **53**: 479–484.](http://paperpile.com/b/rIw3WY/b5Yeo)

10. [Shieh WY, Chen AL, Chiu HH. Vibrio aerogenes sp. nov., a facultatively anaerobic marine bacterium that ferments glucose with gas production. *Int J Syst Evol Microbiol* 2000; **50**: 321–329.](http://paperpile.com/b/rIw3WY/L2d0c)

11. [Wang H, Liu J, Wang Y, Zhang X-H. Vibrio marisflavi sp. nov., isolated from seawater. *Int J Syst Evol Microbiol* 2011; **61**: 568–573.](http://paperpile.com/b/rIw3WY/QEvXK)

12. [Rameshkumar N, Sproer C, Lang E, Nair S. Vibrio mangrovi sp. nov., a diazotrophic bacterium isolated from mangrove-associated wild rice (Poteresia coarctata Tateoka). *FEMS Microbiol Lett* 2010; **307**: 35–40.](http://paperpile.com/b/rIw3WY/BLMTE)

13. [Sheu S-Y, Jiang S-R, Chen CA, Wang J-T, Chen W-M. Vibrio stylophorae sp. nov., isolated from the reef-building coral Stylophora pistillata. *Int J Syst Evol Microbiol* 2011; **61**: 2180–2185.](http://paperpile.com/b/rIw3WY/GG3l9)
