# Supplementary material for: Anaerobic bacterial degradation of protein and lipid macromolecules in subarctic marine sediment
Source: ISME J. 2020 Nov 18;15(3):833–47. doi: 10.1038/s41396-020-00817-6 (PMC8027456; doi:10.1038/s41396-020-00817-6)
Supplement: Supplementary file 2 — Supplementary_Figure_S1 [file 41396_2020_817_MOESM2_ESM.pdf]

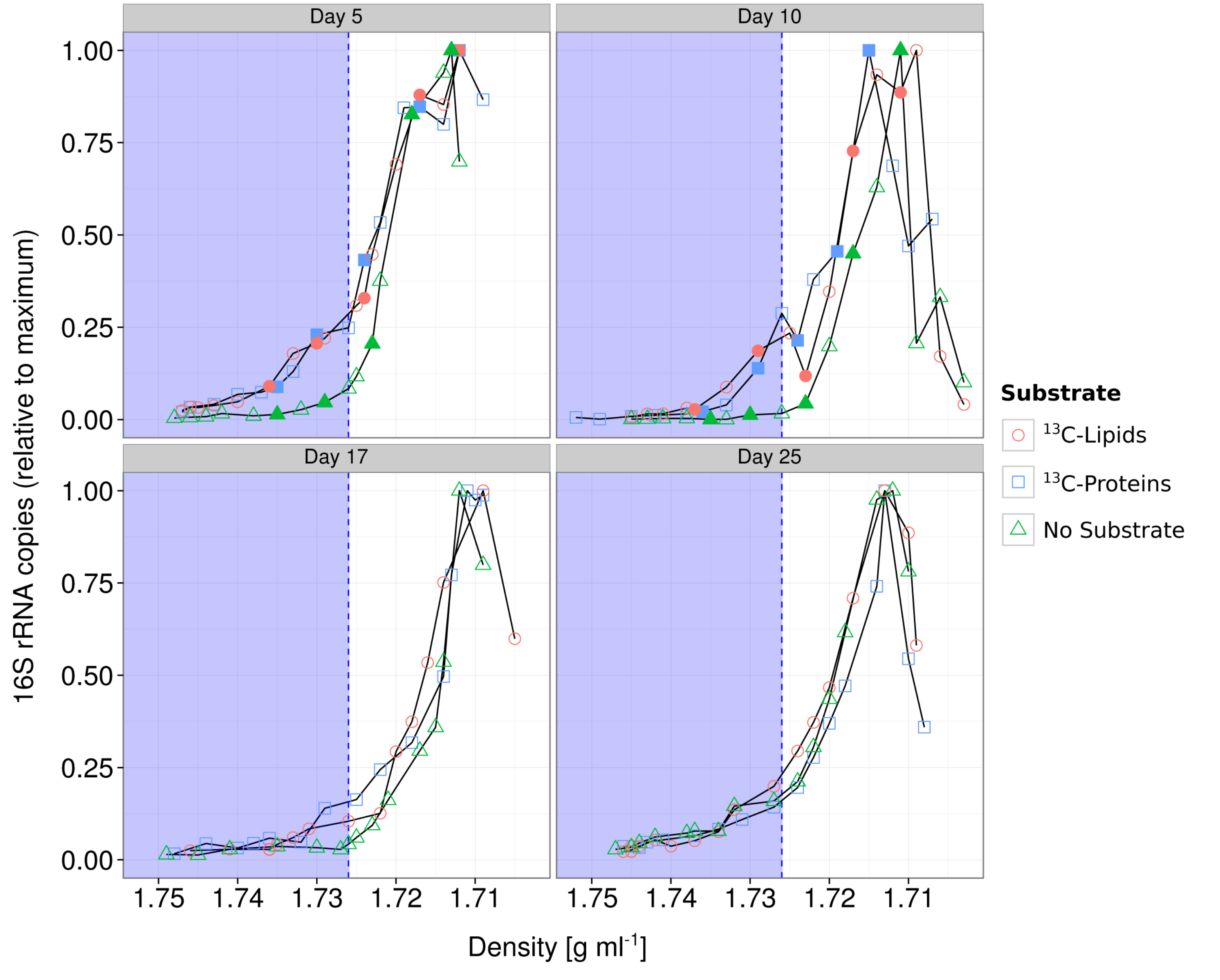

Supplementary Figure S1. Proportions of 16S rRNA gene copies recovered from fractions of DNA-SIP gradients. Color and shape of data points indicate the amended substrate. Filled symbols indicate that DNA from the respective fraction of the DNA-SIP gradient was used as template for amplicon sequencing. The blue shaded area indicates the density of the gradient above which ^{13}C-labelled DNA is expected to accumulate.
