# Supplementary material for: Anaerobic bacterial degradation of protein and lipid macromolecules in subarctic marine sediment
Source: ISME J. 2020 Nov 18;15(3):833–47. doi: 10.1038/s41396-020-00817-6 (PMC8027456; doi:10.1038/s41396-020-00817-6)
Supplement: Supplementary file 3 — Supplementary_Figure_S2 [file 41396_2020_817_MOESM3_ESM.pdf]

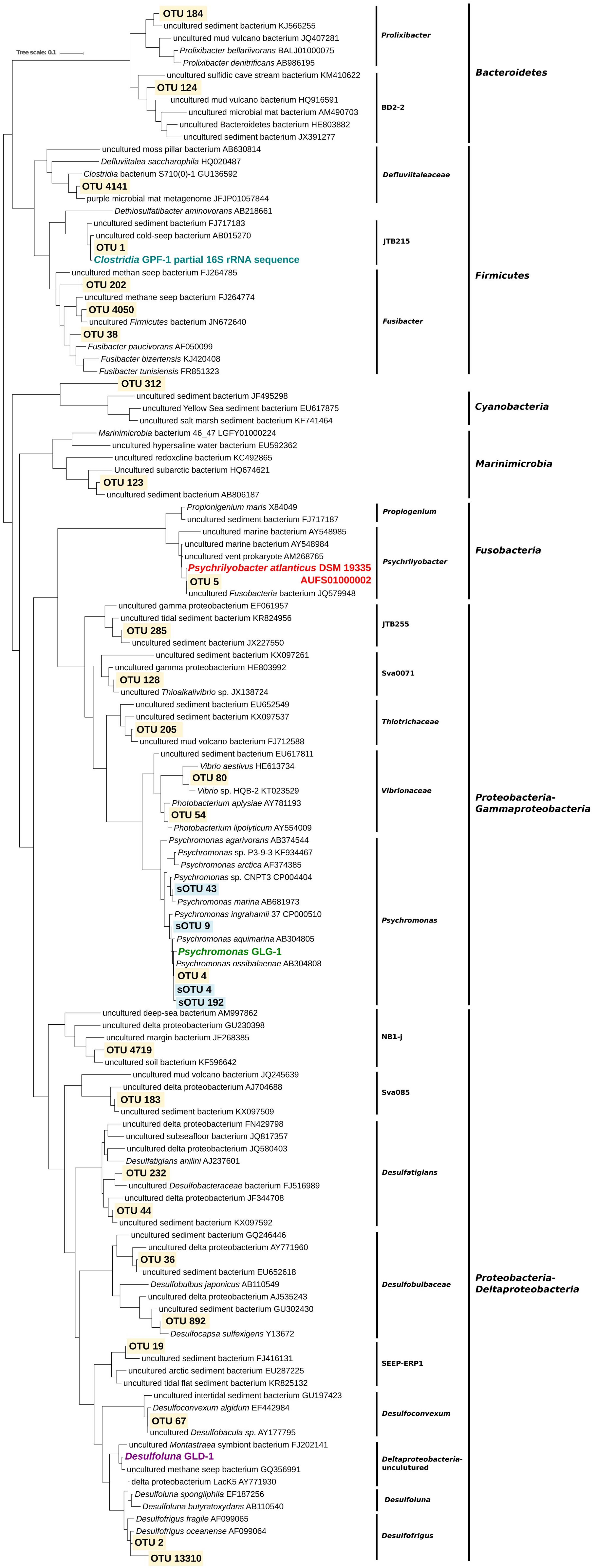

Supplementary Figure S2. Phylogenetic affiliation of 16S rRNA OTU sequences. The tree shows MAGs/genomes and ^{13}C-labelled taxa determined by SIP in relation to close relatives derived from the SILVA database v.128. The color of leaves for the three MAGs and the type strain of *Psychrilyobacter atlanticus* were colored according to the respective color in Figures 4 and 5. The 16S rRNA OTUs and the four *Psychromonas* sub-OTUs were colored yellow and blue, respectively
