# Supplementary material for: Anaerobic bacterial degradation of protein and lipid macromolecules in subarctic marine sediment
Source: ISME J. 2020 Nov 18;15(3):833–47. doi: 10.1038/s41396-020-00817-6 (PMC8027456; doi:10.1038/s41396-020-00817-6)
Supplement: Supplementary file 4 — Supplementary_Figure_S3 [file 41396_2020_817_MOESM4_ESM.pdf]

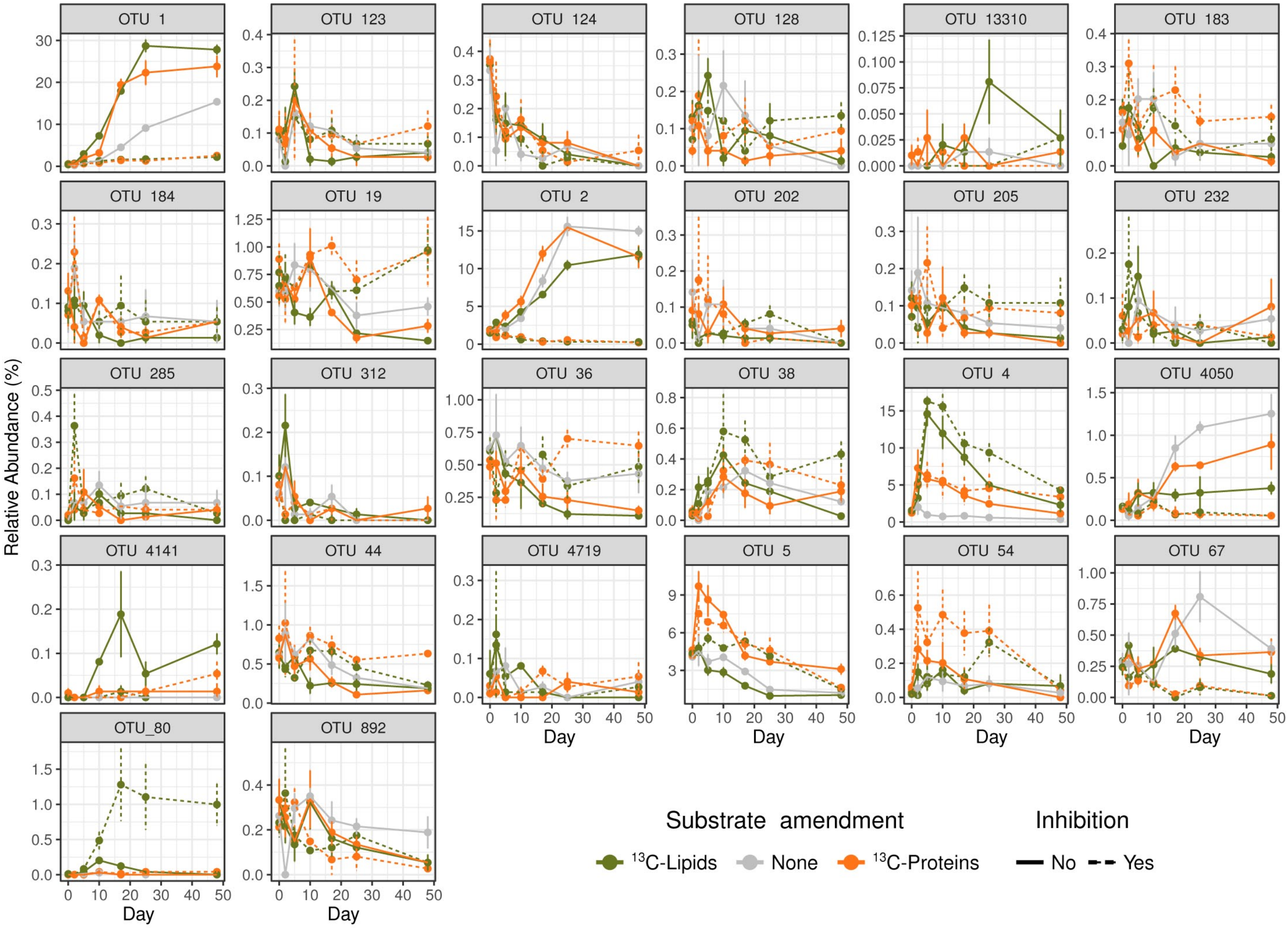

Supplementary Figure S3. Relative abundances of OTUs that incorporated ^{13}C-carbon over-time in from anoxic sediment incubations amended with ^{13}C-proteins or ^{13}C-lipids and from unamended control incubations. OTUs presented correspond to those presented in Figure 2. Inhibition = microcosms where molybdate was added to inhibit sulfate reduction.
