# Supplementary material for: Anaerobic bacterial degradation of protein and lipid macromolecules in subarctic marine sediment
Source: ISME J. 2020 Nov 18;15(3):833–47. doi: 10.1038/s41396-020-00817-6 (PMC8027456; doi:10.1038/s41396-020-00817-6)
Supplement: Supplementary file 5 — Supplementary_Figure_S4 [file 41396_2020_817_MOESM5_ESM.pdf]

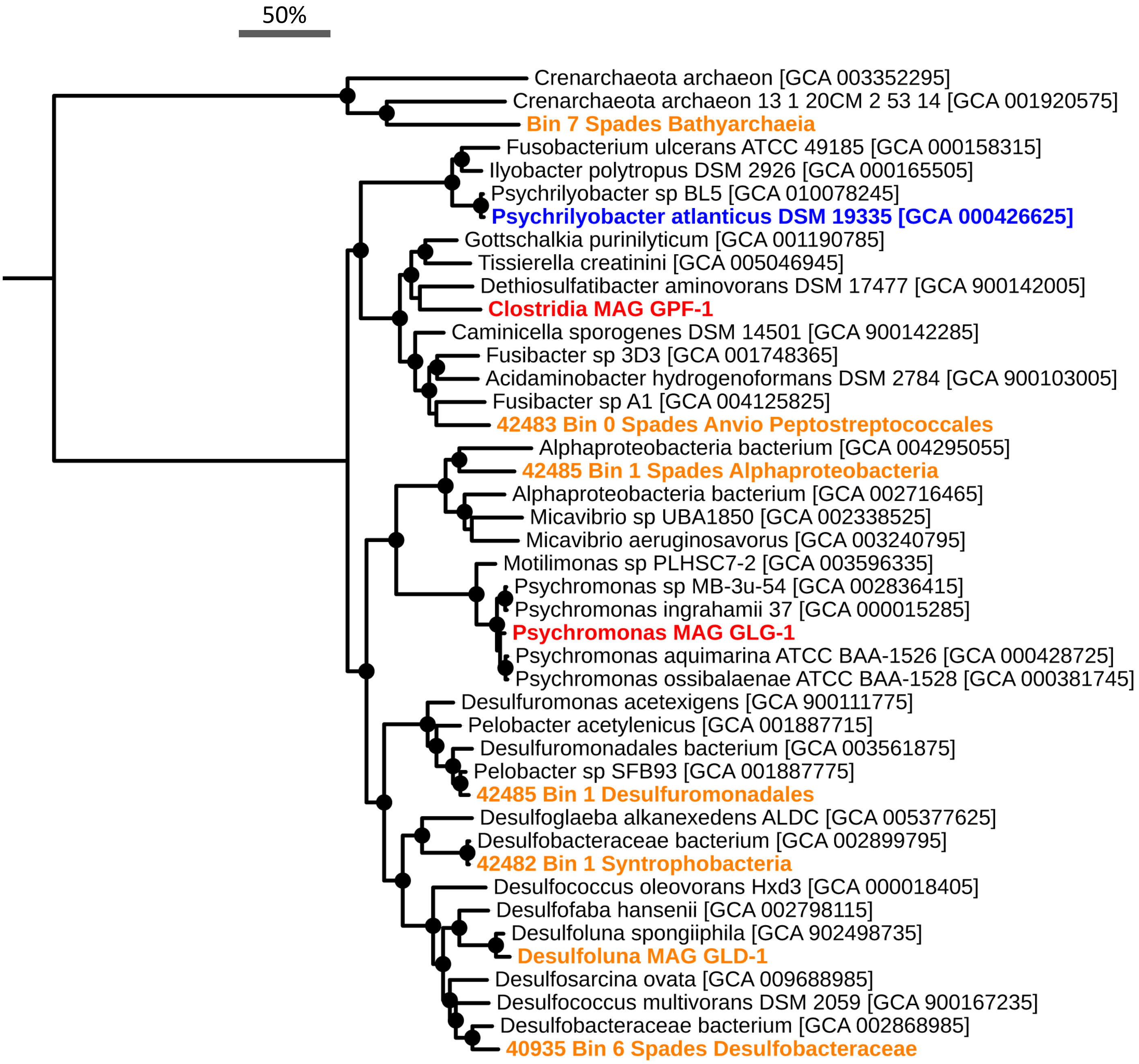

Supplementary Figure S4. Phylogenetic analysis of concatenated single copy marker proteins. The phylogeny (maximum likelihood) is based on concatenated protein sequences derived from single copy marker genes retrieved from CheckM analyses. Red leaves correspond to MAGs of organisms determined to be labelled from DNA-SIP. The blue leaf corresponds to the genome of reference sequence *Psychrilyobacter atlanticus* DSM 19335. Orange leaves correspond to other MAGs recovered in this study. Genbank Bioproject accesssion numbers for MAGs from this study are presented in Supplementary Table S4. Genbank assembly accession numbers for reference genomes are presented in parenthesis. Bootstrap values of >90% are indicated by filled black circles. The scale bar represent 50% sequence divergence.
