# Supplementary material for: Anaerobic bacterial degradation of protein and lipid macromolecules in subarctic marine sediment
Source: ISME J. 2020 Nov 18;15(3):833–47. doi: 10.1038/s41396-020-00817-6 (PMC8027456; doi:10.1038/s41396-020-00817-6)
Supplement: Supplementary file 6 — Supplementary_Figure_S5 [file 41396_2020_817_MOESM6_ESM.pdf]

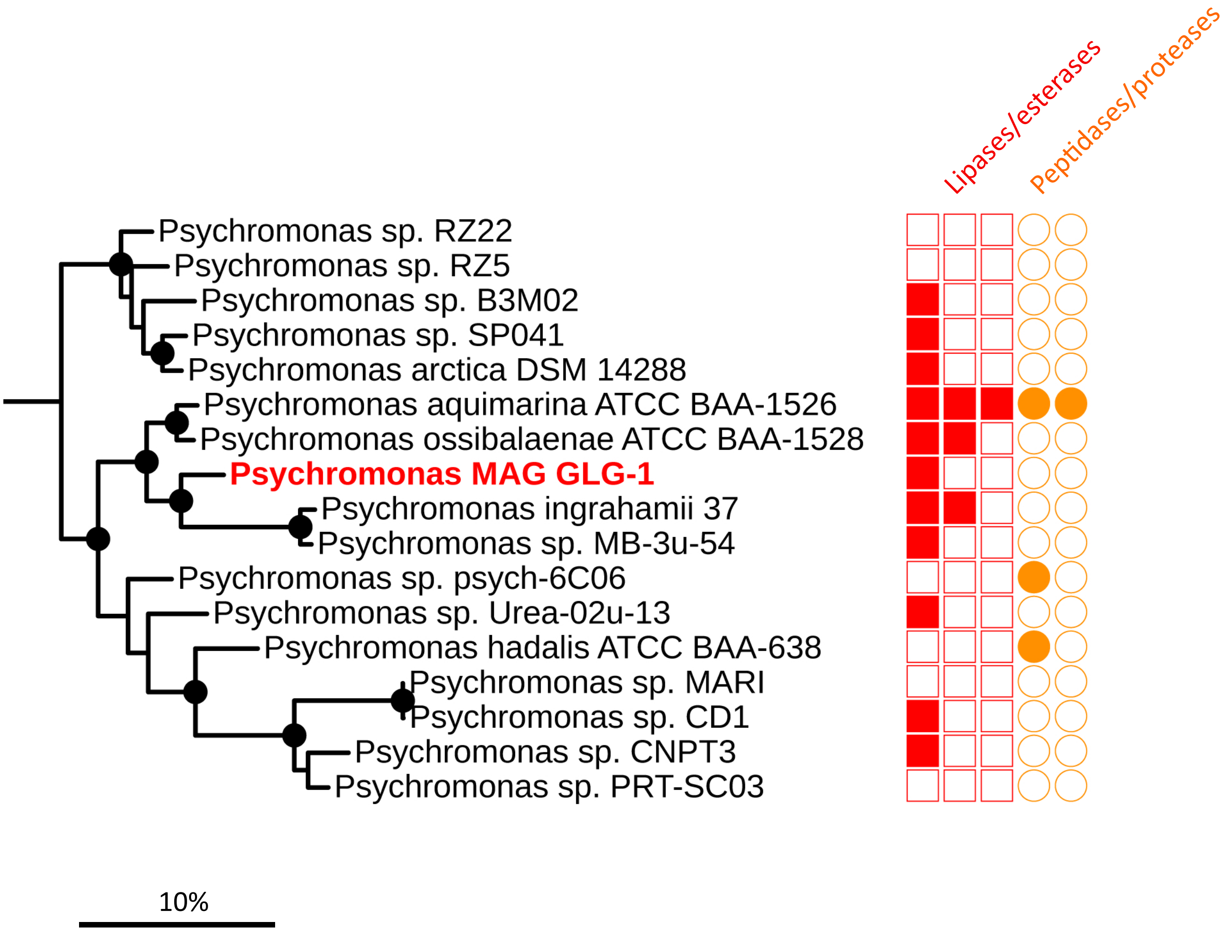

Supplementary Figure S5. Phylogenomic tree of available *Psychromonas* spp. genomes and MAGs, and *Psychromonas* MAG GLG-1 (bold red) recovered in this study. The phylogeny (maximum likelihood) is based on concatenated protein sequences derived from single copy marker genes retrieved from CheckM analyses. Annotations in red or orange correspond to the presence or absence of genes encoding predicted secreted lipase/esterase and peptidase/proteases, respectively (see Supplementary Table 6 for annotations and sequences). Bootstrap values of >90% are indicated by filled black circles. The scale bar represents 10% sequence divergence.
