# Supplementary material for: Anaerobic bacterial degradation of protein and lipid macromolecules in subarctic marine sediment
Source: ISME J. 2020 Nov 18;15(3):833–47. doi: 10.1038/s41396-020-00817-6 (PMC8027456; doi:10.1038/s41396-020-00817-6)
Supplement: Supplementary file 7 — Supplementary_Figure_S6 [file 41396_2020_817_MOESM7_ESM.pdf]

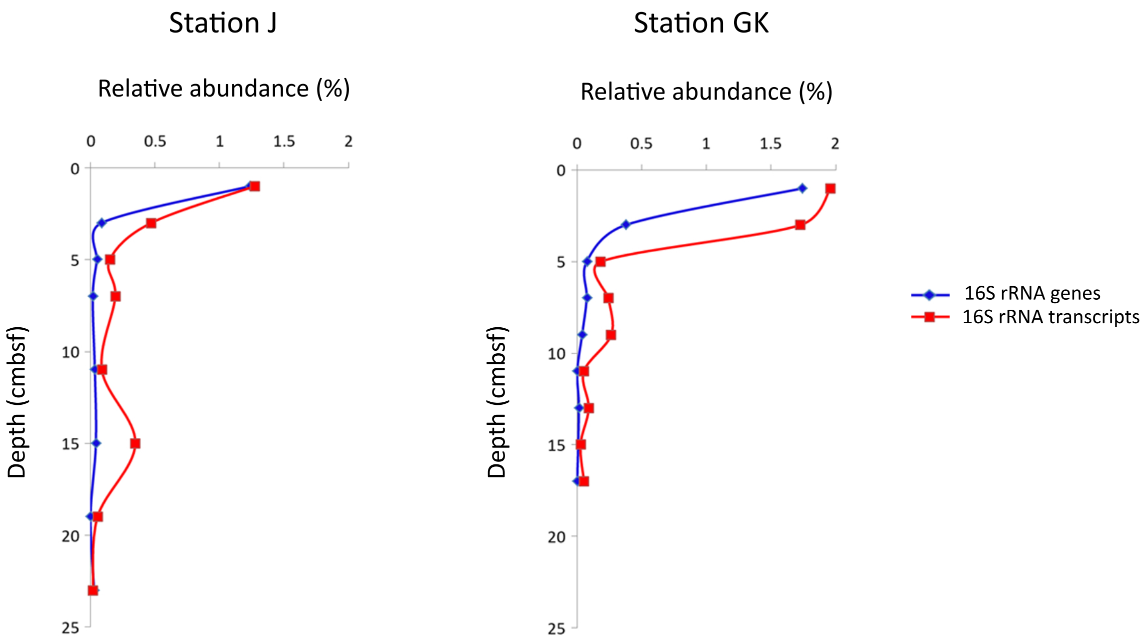

Supplementary Figure S6. Relative abundances of *Psychromonas* spp. in sediments of Smeerenbergfjord, Svalbard, as determined by 16S rRNA-gene and -transcript (cDNA) amplicon sequencing. Cmbsf = Centimeters below seafloor.
