# Supplementary material for: Anaerobic bacterial degradation of protein and lipid macromolecules in subarctic marine sediment
Source: ISME J. 2020 Nov 18;15(3):833–47. doi: 10.1038/s41396-020-00817-6 (PMC8027456; doi:10.1038/s41396-020-00817-6)
Supplement: Supplementary file 8 — Supplementary_Figure_S7 [file 41396_2020_817_MOESM8_ESM.pdf]

(A) *Psychromonas* OTU 4

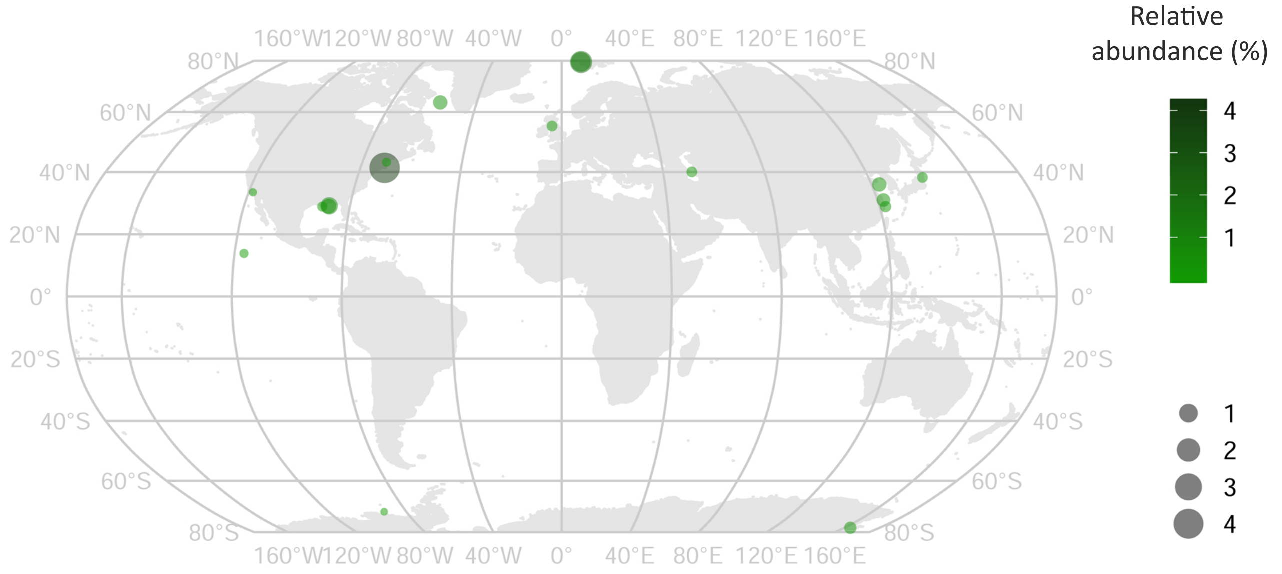

(B) *Psychrilyobacter* OTU 5

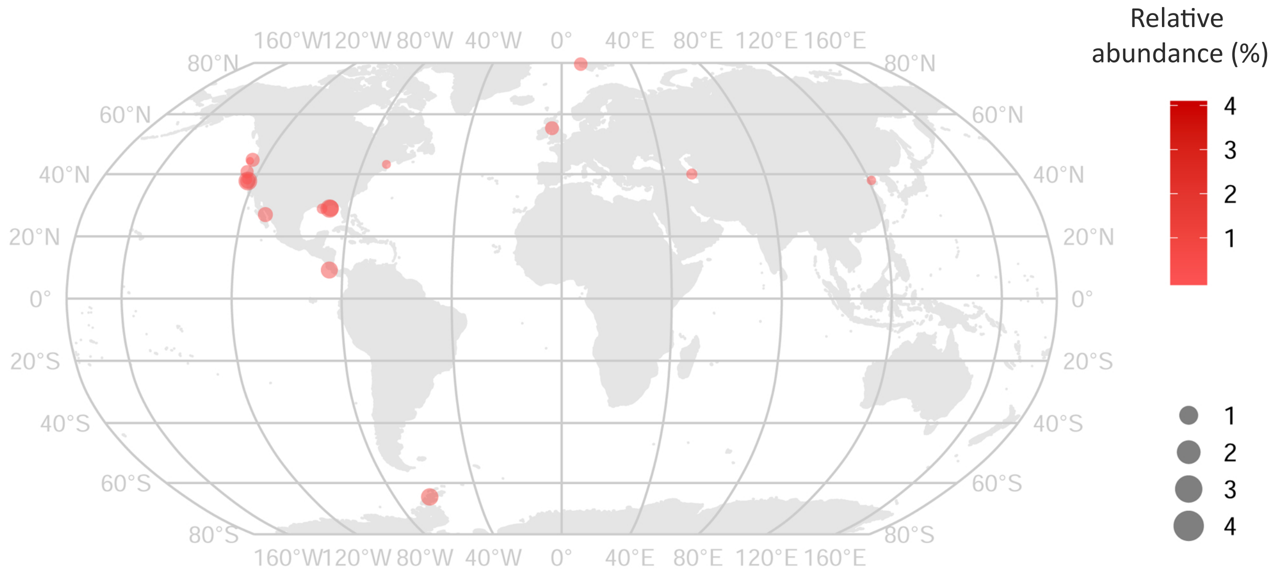

Supplementary Figure S7. Presence and relative abundances of *Psychromonas* OTU 4 (A) *Psychrilyobacter* OTU 5 (B) related sequences (>97%) in publically available 16S rRNA gene datasets, as determined by IMNGS analyses (see Materials and Methods). Additional samples from Svalbard that were determined in this study, are also included.
