# Supplementary material for: Anaerobic bacterial degradation of protein and lipid macromolecules in subarctic marine sediment
Source: ISME J. 2020 Nov 18;15(3):833–47. doi: 10.1038/s41396-020-00817-6 (PMC8027456; doi:10.1038/s41396-020-00817-6)
Supplement: Supplementary file 9 — Supplementary Table S1 [file 41396_2020_817_MOESM9_ESM.pdf]

Supplementary Table S1. Summary of MEROPS and ESTHER database description for peptidases and lipases, respectively.

Peptidases		
**MEROPS ID**	**MEROPS description summary**	**Selected for further analyses**
A8	production of the bacterial cell wall	No
M1	some are cleaving amino acids from small peptides	Yes
M3	Lactobacillus oligoendopeptidase cleaves oligopeptides	Yes
M14	digestion of food and other functions	Yes
M20	conversion of proteins to free amino acids.	Yes
M24	removes N-terminal Methionine from protein	Yes
M28	alkaline phosphatase isozyme conversion	No
M29	catabolic peptidase in Streptococcus thermophilus	Yes
M42	used by Lactobacillus for nutrition on milk-protein powder	Yes
M48	degradation of abnormal proteins	No
M50	regulated intermembrane proetolysis	No
S8	probably involved in nutrition	Yes
S12	synthesis and remodelling of bacterial cell walls	No
S15	degradation of casein	Yes
S24	regulatory stress response	No
S26	remove the signal peptides and facilitate secretion	No

Lipases		
**ESTHER family**	**ESTHER description summary**	**Selected for further analyses**
6_AlphaBeta_hydrolase	not well characterized	No
Abhydrolase_7	acetyl-esterase_deacetylase or Dienelactone_hydrolase or xylan esterase	No
Bacterial_EstLip_FamX	Family of Bacterial lipolytic enzymes	Yes
Carb_B_Bacteria	carboxylesterase, type B	Yes
CarbLipBact_2	members of this group are esterases, lipases	Yes
Duf_1023	proteins of unknown function	No
Duf_3089	lipolytic enzyme family defined from isolation and characterization of two esterases from a metagenomic library	Yes
Duf_900	proteins of unknown function	No
Epoxide_hydrolase	conversion of epoxides to corresponding diols	No
GTSAGmotif	lipases from metagenomic library	Yes
Hormone-sensitive_lipase_like	esterase/lipase, family IV	Yes
Lipase_2	lipases	Yes
Lipase_3	lipases	Yes
Monoglyceridelipase_lysophospholip	has some homology to peptidases S33	No
NFM-deformylase	N-formylmaleamic acid to formic and maleamic acid	No
PGAP1	attachment to proteins factor 1	No
