# Supplementary material for: Anaerobic bacterial degradation of protein and lipid macromolecules in subarctic marine sediment
Source: ISME J. 2020 Nov 18;15(3):833–47. doi: 10.1038/s41396-020-00817-6 (PMC8027456; doi:10.1038/s41396-020-00817-6)
Supplement: Supplementary file 10 — Supplementary Table S2 [file 41396_2020_817_MOESM10_ESM.pdf]

Supplementary Table S2. Statistics results

Day	Comparison	Compound	p_value	adjusted_p_value
Day_0	Proteins vs No_Substrate	Formate_µM	1.000	1.000
Day_2	Proteins vs No_Substrate	Formate_µM	0.043	0.107
Day_5	Proteins vs No_Substrate	Formate_µM	0.483	0.594
Day_10	Proteins vs No_Substrate	Formate_µM	0.401	0.515
Day_17	Proteins vs No_Substrate	Formate_µM	0.247	0.349
Day_0	Proteins vs No_Substrate	Acetate_µM	1.000	1.000
Day_2	Proteins vs No_Substrate	Acetate_µM	0.009	0.035
Day_5	Proteins vs No_Substrate	Acetate_µM	0.005	0.022
Day_10	Proteins vs No_Substrate	Acetate_µM	0.036	0.094
Day_17	Proteins vs No_Substrate	Acetate_µM	0.097	0.185
Day_0	Proteins vs No_Substrate	Propionate_µM	1.000	1.000
Day_2	Proteins vs No_Substrate	Propionate_µM	0.010	0.036
Day_5	Proteins vs No_Substrate	Propionate_µM	0.000	0.002
Day_10	Proteins vs No_Substrate	Propionate_µM	0.223	0.321
Day_17	Proteins vs No_Substrate	Propionate_µM	0.018	0.054
Day_0	Proteins vs No_Substrate	Butyrate_µM	NaN	NaN
Day_2	Proteins vs No_Substrate	Butyrate_µM	0.010	0.035
Day_5	Proteins vs No_Substrate	Butyrate_µM	0.003	0.018
Day_10	Proteins vs No_Substrate	Butyrate_µM	0.598	0.721
Day_17	Proteins vs No_Substrate	Butyrate_µM	0.137	0.232
Day_0	Proteins vs No_Substrate	iso.Butyrate_µM	NaN	NaN
Day_2	Proteins vs No_Substrate	iso.Butyrate_µM	0.003	0.017
Day_5	Proteins vs No_Substrate	iso.Butyrate_µM	0.005	0.022
Day_10	Proteins vs No_Substrate	iso.Butyrate_µM	0.000	0.004
Day_17	Proteins vs No_Substrate	iso.Butyrate_µM	0.067	0.146
Day_0	Proteins vs No_Substrate	Lactate_µM	NaN	NaN
Day_2	Proteins vs No_Substrate	Lactate_µM	NaN	NaN
Day_5	Proteins vs No_Substrate	Lactate_µM	NaN	NaN
Day_10	Proteins vs No_Substrate	Lactate_µM	NaN	NaN
Day_17	Proteins vs No_Substrate	Lactate_µM	NaN	NaN
Day_0	Proteins vs Proteins_inhibited	Formate_µM	1.000	1.000
Day_2	Proteins vs Proteins_inhibited	Formate_µM	0.341	0.448
Day_5	Proteins vs Proteins_inhibited	Formate_µM	0.161	0.258
Day_10	Proteins vs Proteins_inhibited	Formate_µM	0.191	0.283
Day_17	Proteins vs Proteins_inhibited	Formate_µM	0.440	0.556
Day_0	Proteins vs Proteins_inhibited	Acetate_µM	1.000	1.000
Day_2	Proteins vs Proteins_inhibited	Acetate_µM	0.132	0.225
Day_5	Proteins vs Proteins_inhibited	Acetate_µM	0.001	0.010
Day_10	Proteins vs Proteins_inhibited	Acetate_µM	0.000	0.004
Day_17	Proteins vs Proteins_inhibited	Acetate_µM	0.015	0.047
Day_0	Proteins vs Proteins_inhibited	Propionate_µM	1.000	1.000
Day_2	Proteins vs Proteins_inhibited	Propionate_µM	0.005	0.022
Day_5	Proteins vs Proteins_inhibited	Propionate_µM	0.001	0.010
Day_10	Proteins vs Proteins_inhibited	Propionate_µM	0.005	0.022
Day_17	Proteins vs Proteins_inhibited	Propionate_µM	0.001	0.010
Day_0	Proteins vs Proteins_inhibited	Butyrate_µM	NaN	NaN
Day_2	Proteins vs Proteins_inhibited	Butyrate_µM	0.628	0.741
Day_5	Proteins vs Proteins_inhibited	Butyrate_µM	0.044	0.107
Day_10	Proteins vs Proteins_inhibited	Butyrate_µM	0.047	0.110
Day_17	Proteins vs Proteins_inhibited	Butyrate_µM	0.098	0.185
Day_0	Proteins vs Proteins_inhibited	iso.Butyrate_µM	NaN	NaN
Day_2	Proteins vs Proteins_inhibited	iso.Butyrate_µM	0.032	0.090
Day_5	Proteins vs Proteins_inhibited	iso.Butyrate_µM	0.085	0.173
Day_10	Proteins vs Proteins_inhibited	iso.Butyrate_µM	0.851	0.980
Day_17	Proteins vs Proteins_inhibited	iso.Butyrate_µM	0.162	0.258
Day_0	Proteins vs Proteins_inhibited	Lactate_µM	NaN	NaN
Day_2	Proteins vs Proteins_inhibited	Lactate_µM	NaN	NaN
Day_5	Proteins vs Proteins_inhibited	Lactate_µM	NaN	NaN
Day_10	Proteins vs Proteins_inhibited	Lactate_µM	NaN	NaN

Day_17	Proteins vs Proteins_inhibited	Lactate_µM	NaN	NaN
Day_0	Proteins_inhibited vs No Substrate	Formate_µM	1.000	1.000
Day_2	Proteins_inhibited vs No Substrate	Formate_µM	0.183	0.276
Day_5	Proteins_inhibited vs No Substrate	Formate_µM	0.157	0.258
Day_10	Proteins_inhibited vs No Substrate	Formate_µM	0.200	0.293
Day_17	Proteins_inhibited vs No Substrate	Formate_µM	0.174	0.272
Day_0	Proteins_inhibited vs No Substrate	Acetate_µM	1.000	1.000
Day_2	Proteins_inhibited vs No Substrate	Acetate_µM	0.481	0.594
Day_5	Proteins_inhibited vs No Substrate	Acetate_µM	0.037	0.096
Day_10	Proteins_inhibited vs No Substrate	Acetate_µM	0.113	0.209
Day_17	Proteins_inhibited vs No Substrate	Acetate_µM	0.083	0.173
Day_0	Proteins_inhibited vs No Substrate	Propionate_µM	1.000	1.000
Day_2	Proteins_inhibited vs No Substrate	Propionate_µM	0.601	0.721
Day_5	Proteins_inhibited vs No Substrate	Propionate_µM	0.052	0.117
Day_10	Proteins_inhibited vs No Substrate	Propionate_µM	0.393	0.509
Day_17	Proteins_inhibited vs No Substrate	Propionate_µM	0.109	0.204
Day_0	Proteins_inhibited vs No Substrate	Butyrate_µM	NaN	NaN
Day_2	Proteins_inhibited vs No Substrate	Butyrate_µM	0.049	0.115
Day_5	Proteins_inhibited vs No Substrate	Butyrate_µM	0.036	0.094
Day_10	Proteins_inhibited vs No Substrate	Butyrate_µM	0.046	0.110
Day_17	Proteins_inhibited vs No Substrate	Butyrate_µM	0.056	0.124
Day_0	Proteins_inhibited vs No Substrate	iso.Butyrate_µM	NaN	NaN
Day_2	Proteins_inhibited vs No Substrate	iso.Butyrate_µM	0.084	0.173
Day_5	Proteins_inhibited vs No Substrate	iso.Butyrate_µM	0.028	0.081
Day_10	Proteins_inhibited vs No Substrate	iso.Butyrate_µM	0.087	0.173
Day_17	Proteins_inhibited vs No Substrate	iso.Butyrate_µM	0.089	0.173
Day_0	Proteins_inhibited vs No Substrate	Lactate_µM	NaN	NaN
Day_2	Proteins_inhibited vs No Substrate	Lactate_µM	NaN	NaN
Day_5	Proteins_inhibited vs No Substrate	Lactate_µM	NaN	NaN
Day_10	Proteins_inhibited vs No Substrate	Lactate_µM	NaN	NaN
Day_17	Proteins_inhibited vs No Substrate	Lactate_µM	NaN	NaN
Day_0	Lipids vs No_Substrate	Formate_µM	1.000	1.000
Day_2	Lipids vs No_Substrate	Formate_µM	0.163	0.258
Day_5	Lipids vs No_Substrate	Formate_µM	0.271	0.372
Day_10	Lipids vs No_Substrate	Formate_µM	0.342	0.448
Day_17	Lipids vs No_Substrate	Formate_µM	0.284	0.382
Day_0	Lipids vs No_Substrate	Acetate_µM	1.000	1.000
Day_2	Lipids vs No_Substrate	Acetate_µM	0.119	0.211
Day_5	Lipids vs No_Substrate	Acetate_µM	0.127	0.220
Day_10	Lipids vs No_Substrate	Acetate_µM	0.114	0.209
Day_17	Lipids vs No_Substrate	Acetate_µM	0.444	0.556
Day_0	Lipids vs No_Substrate	Propionate_µM	1.000	1.000
Day_2	Lipids vs No_Substrate	Propionate_µM	0.036	0.094
Day_5	Lipids vs No_Substrate	Propionate_µM	0.159	0.258
Day_10	Lipids vs No_Substrate	Propionate_µM	0.270	0.372
Day_17	Lipids vs No_Substrate	Propionate_µM	0.085	0.173
Day_0	Lipids vs No_Substrate	Butyrate_µM	NaN	NaN
Day_2	Lipids vs No_Substrate	Butyrate_µM	0.042	0.107
Day_5	Lipids vs No_Substrate	Butyrate_µM	0.002	0.014
Day_10	Lipids vs No_Substrate	Butyrate_µM	0.016	0.051
Day_17	Lipids vs No_Substrate	Butyrate_µM	0.018	0.054
Day_0	Lipids vs No_Substrate	iso.Butyrate_µM	NaN	NaN
Day_2	Lipids vs No_Substrate	iso.Butyrate_µM	0.017	0.053
Day_5	Lipids vs No_Substrate	iso.Butyrate_µM	0.013	0.044
Day_10	Lipids vs No_Substrate	iso.Butyrate_µM	0.002	0.015
Day_17	Lipids vs No_Substrate	iso.Butyrate_µM	0.008	0.033
Day_0	Lipids vs No_Substrate	Lactate_µM	NaN	NaN
Day_2	Lipids vs No_Substrate	Lactate_µM	NaN	NaN
Day_5	Lipids vs No_Substrate	Lactate_µM	NaN	NaN
Day_10	Lipids vs No_Substrate	Lactate_µM	NaN	NaN
Day_17	Lipids vs No_Substrate	Lactate_µM	NaN	NaN
Day_0	Lipids vs Lipids_inhibited	Formate_µM	1.000	1.000

Day_2	Lipids vs Lipids_inhibited	Formate_µM	0.235	0.335
Day_5	Lipids vs Lipids_inhibited	Formate_µM	0.001	0.010
Day_10	Lipids vs Lipids_inhibited	Formate_µM	0.003	0.018
Day_17	Lipids vs Lipids_inhibited	Formate_µM	0.000	0.004
Day_0	Lipids vs Lipids_inhibited	Acetate_µM	1.000	1.000
Day_2	Lipids vs Lipids_inhibited	Acetate_µM	0.119	0.211
Day_5	Lipids vs Lipids_inhibited	Acetate_µM	0.074	0.159
Day_10	Lipids vs Lipids_inhibited	Acetate_µM	0.000	0.002
Day_17	Lipids vs Lipids_inhibited	Acetate_µM	0.011	0.036
Day_0	Lipids vs Lipids_inhibited	Propionate_µM	1.000	1.000
Day_2	Lipids vs Lipids_inhibited	Propionate_µM	0.259	0.362
Day_5	Lipids vs Lipids_inhibited	Propionate_µM	0.295	0.394
Day_10	Lipids vs Lipids_inhibited	Propionate_µM	0.052	0.117
Day_17	Lipids vs Lipids_inhibited	Propionate_µM	0.120	0.211
Day_0	Lipids vs Lipids_inhibited	Butyrate_µM	NaN	NaN
Day_2	Lipids vs Lipids_inhibited	Butyrate_µM	0.994	1.000
Day_5	Lipids vs Lipids_inhibited	Butyrate_µM	0.001	0.010
Day_10	Lipids vs Lipids_inhibited	Butyrate_µM	0.002	0.015
Day_17	Lipids vs Lipids_inhibited	Butyrate_µM	0.001	0.010
Day_0	Lipids vs Lipids_inhibited	iso.Butyrate_µM	NaN	NaN
Day_2	Lipids vs Lipids_inhibited	iso.Butyrate_µM	0.223	0.321
Day_5	Lipids vs Lipids_inhibited	iso.Butyrate_µM	0.009	0.034
Day_10	Lipids vs Lipids_inhibited	iso.Butyrate_µM	0.001	0.010
Day_17	Lipids vs Lipids_inhibited	iso.Butyrate_µM	0.013	0.042
Day_0	Lipids vs Lipids_inhibited	Lactate_µM	NaN	NaN
Day_2	Lipids vs Lipids_inhibited	Lactate_µM	0.184	0.276
Day_5	Lipids vs Lipids_inhibited	Lactate_µM	0.004	0.020
Day_10	Lipids vs Lipids_inhibited	Lactate_µM	0.004	0.021
Day_17	Lipids vs Lipids_inhibited	Lactate_µM	NaN	NaN
Day_0	Lipids_inhibited vs No Substrate	Formate_µM	1.000	1.000
Day_2	Lipids_inhibited vs No Substrate	Formate_µM	0.184	0.276
Day_5	Lipids_inhibited vs No Substrate	Formate_µM	0.001	0.010
Day_10	Lipids_inhibited vs No Substrate	Formate_µM	0.003	0.018
Day_17	Lipids_inhibited vs No Substrate	Formate_µM	0.001	0.010
Day_0	Lipids_inhibited vs No Substrate	Acetate_µM	1.000	1.000
Day_2	Lipids_inhibited vs No Substrate	Acetate_µM	0.436	0.556
Day_5	Lipids_inhibited vs No Substrate	Acetate_µM	0.033	0.091
Day_10	Lipids_inhibited vs No Substrate	Acetate_µM	0.163	0.258
Day_17	Lipids_inhibited vs No Substrate	Acetate_µM	0.089	0.173
Day_0	Lipids_inhibited vs No Substrate	Propionate_µM	1.000	1.000
Day_2	Lipids_inhibited vs No Substrate	Propionate_µM	0.635	0.744
Day_5	Lipids_inhibited vs No Substrate	Propionate_µM	0.000	0.004
Day_10	Lipids_inhibited vs No Substrate	Propionate_µM	0.720	0.836
Day_17	Lipids_inhibited vs No Substrate	Propionate_µM	0.533	0.650
Day_0	Lipids_inhibited vs No Substrate	Butyrate_µM	NaN	NaN
Day_2	Lipids_inhibited vs No Substrate	Butyrate_µM	0.279	0.380
Day_5	Lipids_inhibited vs No Substrate	Butyrate_µM	0.001	0.010
Day_10	Lipids_inhibited vs No Substrate	Butyrate_µM	0.002	0.016
Day_17	Lipids_inhibited vs No Substrate	Butyrate_µM	0.002	0.016
Day_0	Lipids_inhibited vs No Substrate	iso.Butyrate_µM	NaN	NaN
Day_2	Lipids_inhibited vs No Substrate	iso.Butyrate_µM	0.610	0.726
Day_5	Lipids_inhibited vs No Substrate	iso.Butyrate_µM	0.007	0.029
Day_10	Lipids_inhibited vs No Substrate	iso.Butyrate_µM	0.000	0.007
Day_17	Lipids_inhibited vs No Substrate	iso.Butyrate_µM	0.009	0.033
Day_0	Lipids_inhibited vs No Substrate	Lactate_µM	NaN	NaN
Day_2	Lipids_inhibited vs No Substrate	Lactate_µM	0.184	0.276
Day_5	Lipids_inhibited vs No Substrate	Lactate_µM	0.004	0.020
Day_10	Lipids_inhibited vs No Substrate	Lactate_µM	0.004	0.021
Day_17	Lipids_inhibited vs No Substrate	Lactate_µM	NaN	NaN
