# Supplementary material for: Anaerobic bacterial degradation of protein and lipid macromolecules in subarctic marine sediment
Source: ISME J. 2020 Nov 18;15(3):833–47. doi: 10.1038/s41396-020-00817-6 (PMC8027456; doi:10.1038/s41396-020-00817-6)
Supplement: Supplementary file 11 — Supplementary Table S3 [file 41396_2020_817_MOESM11_ESM.pdf]

Supplementary Table S3. Significant enrichment of 16S rRNA gene OTUs in ^{13}C fractions of DNA stable isotope gradients.

OTU	Taxonomy	base mean	log2-fold change	adjusted p-value	Treatment	Day
OTU_80	*Gammaproteobacteria* , *Vibrionaceae*	8.7	7.7	0.000	Proteins	10
OTU_80	Gammaproteobacteria, Vibrionaceae	8.7	6.7	0.002	Lipids	5
OTU_232	*Deltaproteobacteria* , *Desulfatiglans*	2.4	6.4	0.005	Proteins	10
OTU_4	*Gammaproteobacteria* , *Psychromonas*	936.5	5.5	0.000	Lipids	5
OTU_312	*Cyanobacteria*	1.9	5.2	0.034	Proteins	10
OTU_4	*Gammaproteobacteria* , *Psychromonas*	936.5	4.9	0.000	Lipids	10
OTU_4141	*Firmicutes* , *Defluviitaleaceae*	5.2	4.8	0.015	Lipids	10
OTU_183	*Deltaproteobacteria* , Sva0485	4.2	4.8	0.003	Proteins	10
OTU_184	*Bacteroidetes* , *Prolixibacter*	1.4	4.5	0.083	Proteins	10
OTU_123	*Marinimicrobia*	4.3	4.5	0.007	Proteins	10
OTU_4	*Gammaproteobacteria* , *Psychromonas*	936.5	4.5	0.000	Proteins	5
OTU_80	*Gammaproteobacteria* , *Vibrionaceae*	8.7	4.3	0.015	Lipids	10
OTU_124	*Bacteroidetes* , BD2-2	3.6	3.7	0.055	Proteins	10
OTU_285	*Gammaproteobacteria* , JTB255	2.7	3.6	0.083	Proteins	10
OTU_13310	*Deltaproteobacteria* , *Desulfofrigus*	5.4	3.5	0.072	Lipids	10
OTU_4719	*Deltaproteobacteria* , NB1-J	3.1	3.3	0.098	Proteins	10
OTU_54	*Gammaproteobacteria* , *Vibrionaceae*	9.1	3.2	0.030	Proteins	5
OTU_128	*Gammaproteobacteria* , Sva0071	4.1	3.1	0.045	Proteins	10
OTU_1	*Firmicutes* , JTB215	967.1	3.0	0.018	Proteins	5
OTU_202	*Firmicutes* , *Clostridiales*	2.1	3.0	0.098	Proteins	10
OTU_205	*Gammaproteobacteria* , Thiotrichaceae	3.2	3.0	0.083	Proteins	10
OTU_4050	*Firmicutes* , *Fusibacter*	25.8	3.0	0.001	Proteins	10
OTU_4	*Gammaproteobacteria* , *Psychromonas*	936.5	2.8	0.009	Proteins	10
OTU_1	*Firmicutes* , JTB215	967.1	2.8	0.036	Lipids	10
OTU_38	*Firmicutes* , *Clostridiales*	11.2	2.6	0.006	Proteins	5
OTU_19	*Deltaproteobacteria* , SEEP-SRB1	22.5	2.1	0.009	Proteins	10
OTU_892	*Deltaproteobacteria* , *Desulfobulbaceae*	11.0	2.1	0.098	Proteins	10
OTU_5	*Fusobacteria* , *Psychrilyobacter*	297.5	2.0	0.005	Proteins	5
OTU_36	*Deltaproteobacteria* , *Desulfobulbaceae*	16.0	2.0	0.068	Proteins	10
OTU_44	*Deltaproteobacteria* , *Desulfatiglans*	16.5	1.8	0.098	Proteins	10
OTU_67	*Deltaproteobacteria* , *Desulfoconvexum*	19.0	1.7	0.055	Proteins	10
OTU_2	*Deltaproteobacteria* , *Desulfofrigus*	699.6	1.3	0.043	Proteins	10
OTU_5	*Fusobacteria* , *Psychrilyobacter*	297.5	1.3	0.055	Proteins	10
