# Supplementary material for: Anaerobic bacterial degradation of protein and lipid macromolecules in subarctic marine sediment
Source: ISME J. 2020 Nov 18;15(3):833–47. doi: 10.1038/s41396-020-00817-6 (PMC8027456; doi:10.1038/s41396-020-00817-6)
Supplement: Supplementary file 12 — Supplementary Table S4 [file 41396_2020_817_MOESM12_ESM.pdf]

Supplementary Table S4. Genome statistics of metagenome-assembled genomes.

MAG	Genbank accession no.	Microscope annotation ID	GTDB classification	Closest relative [ANI (% aligned) / AAI (% aligned)]	Complete-ness (%)	Contam-ination (%)	Strain heterog-eneity	Metagenome bin origin
Psychromonas GLG-1	SAMN14421524	42481_Bin_4	d__Bacteria;p__Proteobacteria;c__Gammaproteobacteria;o__Enterobacterales;f__Psychromonadaceae;g__Psychromonas;s_	*Psychromonas aquimarina* ATCC BAA-1526 GCF_000381745.1 [80.7 (41.8) / 80.73 (66.28)]	97.39	0.81	50	Lipid microcosm day 5
Clostridia GPF-1	SAMN14421525	42482_Bin_3	d__Bacteria;p__Firmicutes_A;c__Clostridia;o__Tissierellales;f__;g__;s__	*Caloranaerobacter azorensis* DSM 13643 GCA_900129995.1 [- / 53.39 (37)]	99.07	0.7	0	Protein microcosm day 17
Desulfoluna GLD-1	SAMN14421526	42483_Bin_2	d__Bacteria;p__Desulfobacterota;c__Desulfobacteria;o__Desulfobacterales;f__Desulfobacteraceae;g__Desulfoluna;s__	*Desulfoluna spongiiphila* GCA_900101345.1 [79.42 (39.41) / 76.93 (68.72)]	94.95	1.36	0	Lipid microcosm day 17
42485_Bin_1	SAMN14421527	.	d__Bacteria;p__Desulfuromonadota;c__Desulfuromonadia;o__Desulfuromonadales;f__Pelobacteraceae_A;g__SFB93;s__	*Pelobacter* sp. SFB93 GCF_001887775.1 [80.99 (60.65) / 84.98 (67.80)]	98.71	1.47	14.29	Lipid microcosm day 25
42482_Bin_1	SAMN14421528	.	d__Bacteria;p__Desulfobacterota;c__Syntrophobacteria;o__BM002;f__BM002;g__BM002;s__BM002 sp002899795	*Desulfobacteraceae* bacterium GCA_002899795.1 [95.31 (73.68) / 95.26 (64.16)]	96.61	3.39	0	Protein microcosm day 17
40935_Bin_6_Spades	SAMN14421529	.	d__Bacteria;p__Desulfobacterota;c__Desulfobacteria;o__Desulfobacterales;f__BuS5;g__;s__	*Desulfobacteraceae* bacterium GCA_002868985.1 [79.31 (38.02) / 76.48 (45.11)]	87.49	2.71	30	Lipid microcosm day 25
42483_Bin_0_Spades_Anvio	SAMN14421530	.	d__Bacteria;p__Firmicutes_A;c__Clostridia;o__Peptostreptococcales;f__;g__;s__	*Caminicella sporogenes* DSM 14501 GCF_900142285.1 [- / 56.06 (42.81)]	85.99	4.25	0	Lipid microcosm day 17
40935_Bin_7_Spades	SAMN14421531	.	d__Archaea;p__Crenarchaeota;c__Bathyarchaeia;o__TCS64;f__TCS64;g__RBG-16-57-9;s__	Candidatus *Bathyarchaeota* archaeon GCA_004525915.1 [77.69 (15.99) / 71.11 (51.03)]	83.64	3.74	0	Lipid microcosm day 25
42485_Bin_1_Spades	SAMN14421532	.	d__Bacteria;p__Proteobacteria;c__Alphaproteobacteria;o__Micavibrionales;f__;g__;s__	*Azospirillum* sp. K2W22B-5 GCF_003590795.1 [- / 55.67 (43.89)]	80.7	0.43	0	Lipid microcosm day 25
