# Supplementary material for: Anaerobic bacterial degradation of protein and lipid macromolecules in subarctic marine sediment
Source: ISME J. 2020 Nov 18;15(3):833–47. doi: 10.1038/s41396-020-00817-6 (PMC8027456; doi:10.1038/s41396-020-00817-6)
Supplement: Supplementary file 14 — Supplementary Table S6 [file 41396_2020_817_MOESM14_ESM.pdf]

Supplementary Table S6. Summary of additional *Psychromonas* genomes and MAGs, and key annotations for predicted secreted lipases/esterases and peptidases/proteases.

Genome	Genbank assembly accession no.	Sequence size (bp)	No. of contigs	GC (%)	Complete-ness	Contam-ination	Strain hetero-geneity	RAST annotation	PsortB location prediction	CDD domain (NCBI)
Psychromonas ossibalaenae ATCC BAA-1528	GCA_000381745	5202369	107	42	100	0	0	peg.1148 Phosphodiesterase/alkaline phosphatase D	Extracellular	Phosphodiesterase/alkaline phosphatase D, PhoD
								peg.1957 Thermolabile hemolysin precursor	Extracellular	SGNH_hydrolase
Psychromonas aquimarina ATCC BAA-1526	GCA_000428725	5534630	141	42.5	100	0	0	peg.1078 Lipase precursor (EC 3.1.1.3)	Extracellular	Triacylglycerol esterase/lipase EstA
								peg.2731 thermolabile hemolysin	Extracellular	GDSL-like Lipase/Acylhydrolase
								peg.3736 Thermolabile hemolysin precursor	Extracellular	Phosphodiesterase/alkaline phosphatase D, PhoD
								peg.3412 Alkaline serine protease	Extracellular	Gluzincin Peptidase family (thermolysin-like proteinases
								peg.4597 secreted trypsin-like serine protease	Extracellular	Trypsin-like serine protease
Psychromonas ingrahamii 37	GCA_000015285	4559598	1	40.1	100	0.15	60	peg.3001 Phosphodiesterase/alkaline phosphatase D	Extracellular	Phosphodiesterase/alkaline phosphatase D, PhoD
								peg.1920 Phospholipase/lecithinase/hemolysin	Extracellular	SGNH_hydrolase
Psychromonas arctica DSM 14288	GCA_000482725	4745897	81	37.8	99.46	0.75	66.67	peg.2566 Phosphodiesterase/alkaline phosphatase D	Extracellular	Phosphodiesterase/alkaline phosphatase D, PhoD
Psychromonas hadalis ATCC BAA-638	GCA_000420245	3979980	351	39.1	100	0.54	0	peg.25 peptidase S8 and S53, subtilisin	Extracellular	Peptidase S8 family
Psychromonas sp. CNPT3	GCA_000153405	3052410	1	38.6	100	0	0	peg.2370 Thermolabile hemolysin precursor	Extracellular	COG3240 Phospholipase
Psychromonas sp. CD1	GCA_002239585	1688234	3	36.9	91.98	0	0	peg.1338 Thermolabile hemolysin precursor	Extracellular	COG3240 Phospholipase
Psychromonas sp. psych-6C06	GCA_002835465	3719886	32	39.3	100	0.09	0	peg.1896 Vibriolysin, extracellular zinc protease	Extracellular	Gluzincin Peptidase family (thermolysin-like proteinases
Psychromonas sp. RZ22	GCA_004376845	3069985	38	35.9	100	0	0	.	.	.
Psychromonas sp. SP041	GCA_000470315	4801754	121	36.4	100	7.21	28	peg.2919 Phosphodiesterase/alkaline phosphatase D	Extracellular	Phosphodiesterase/alkaline phosphatase D, PhoD
Psychromonas sp. RZ5	GCA_004378355	3511338	116	36.7	100	0.63	0	.	.	.
Psychromonas sp. Urea-02u-13	GCA_002835995	4749264	320	39	100	0.23	83.33	peg.584 Alkaline serine protease	Extracellular	Zinc-dependent metalloprotease
Psychromonas sp. MB-3u-54	GCA_002836415	4371403	186	40.8	100	1.02	28.57	peg. 837 Phospholipase/lecithinase/hemolysin	Extracellular	SGNH_hydrolase
Psychromonas sp. B3M02	GCA_003318165	3861573	156	38.4	100	0	0	peg.969 Phosphodiesterase/alkaline phosphatase D	Extracellular	Phosphodiesterase/alkaline phosphatase D, PhoD
Psychromonas sp. PRT-SC03	GCA_001321825	906655	210	37.1	30.45	0.86	66.67	None, although incomplete genome	.	.
Psychromonas sp. MARI	GCA_002848885	1487260	464	36.9	84.52	2.16	40	None, although incomplete genome	.	.
